# Supplementary material for: Intercalant-independent transition temperature in superconducting black phosphorus
Source: Nat Commun. 2017 Apr 12;8:15036. doi: 10.1038/ncomms15036 (PMC5394339; doi:10.1038/ncomms15036)
Supplement: Supplementary Information — Supplementary Figures [file ncomms15036-s1.pdf]

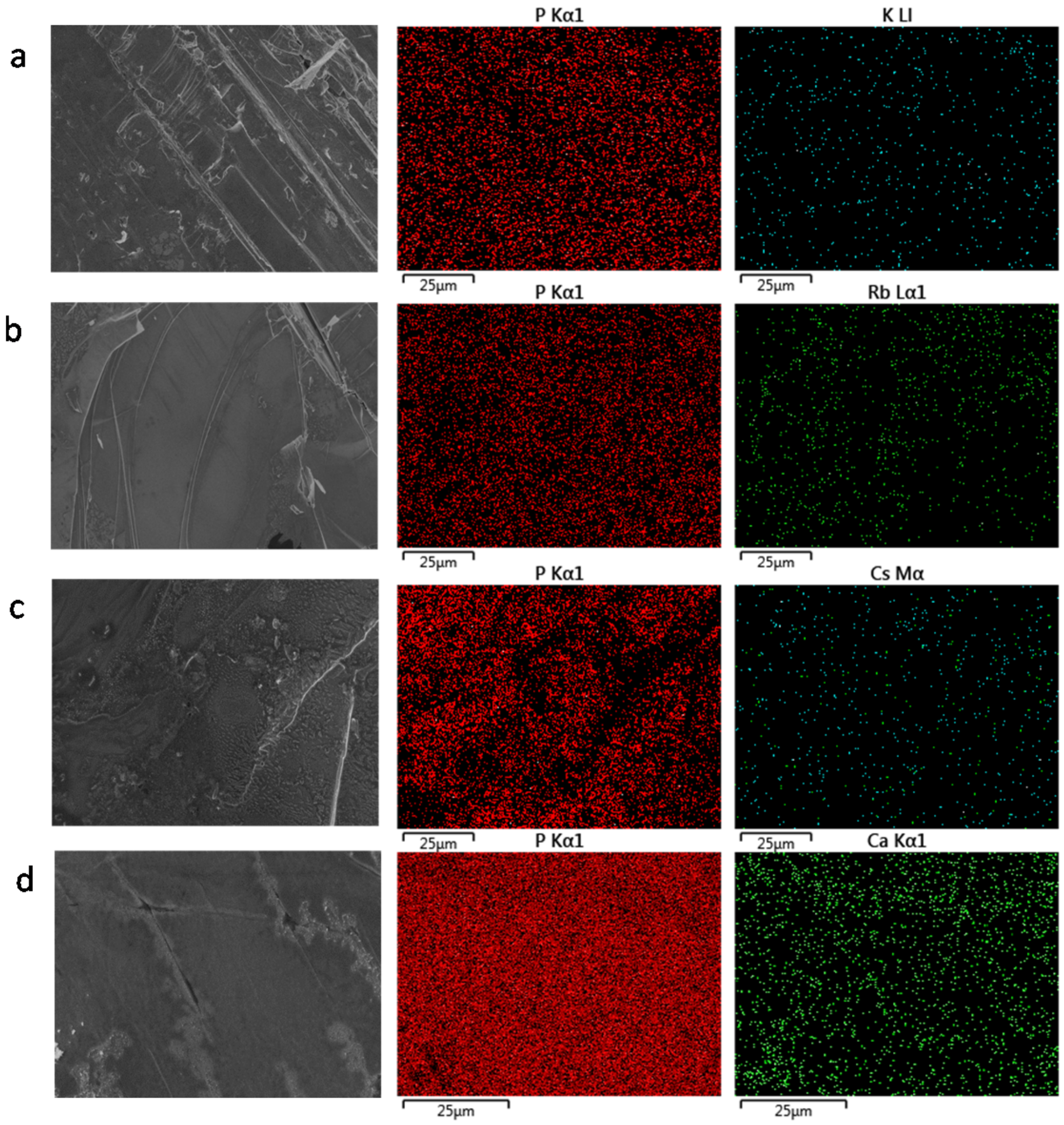

Supplementary Figure 1 | Distribution of metal dopants in intercalated black phosphorus from EDS analysis. (a-d) Shown are SEM images (left column) and corresponding elemental maps for K-, Rb-, Cs- and Ca-intercalated black phosphorus, respectively. For all intercalated samples, the distribution of intercalants follows closely the shape of the crystals as seen from the distribution of P atoms.

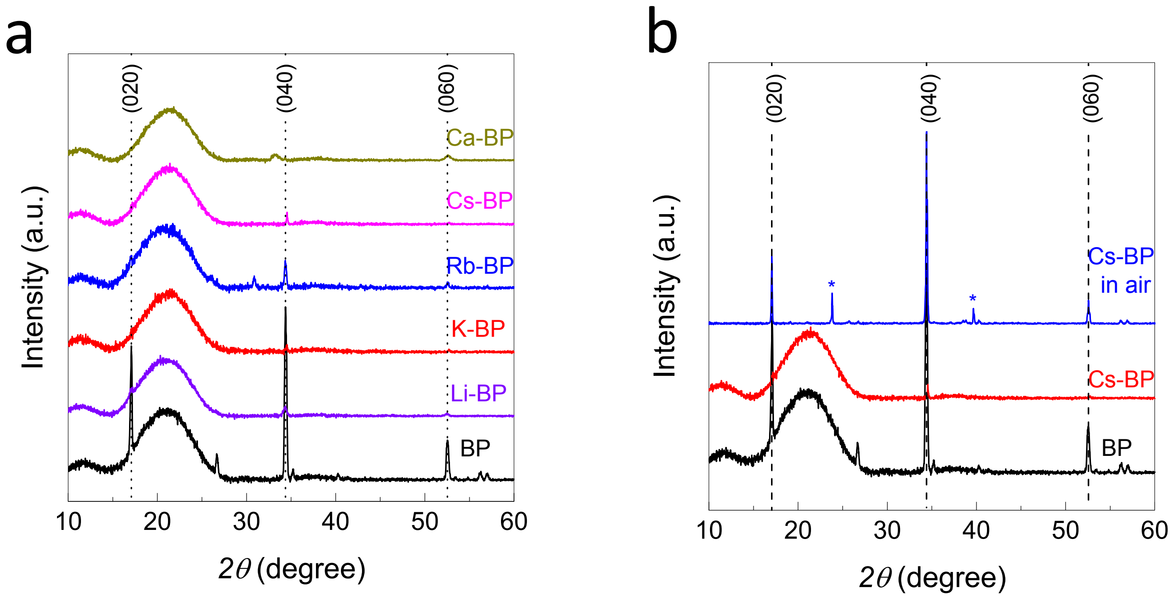

Supplementary Figure 2 | X-ray diffraction spectra for pristine, intercalated and de-intercalated black phosphorus. (a) XRD spectra for pristine and intercalated BP. Dashed lines indicate known positions of (020), (040) and (060) peaks for pristine BP. **(b)** Comparison of XRD spectra for pristine BP (black curve), Cs-intercalated BP (red curve) and the same Cs-intercalated sample that was allowed to de-intercalate by exposure to air for 100 hours (blue curve). * indicates the known peaks for Cs hydroxide.

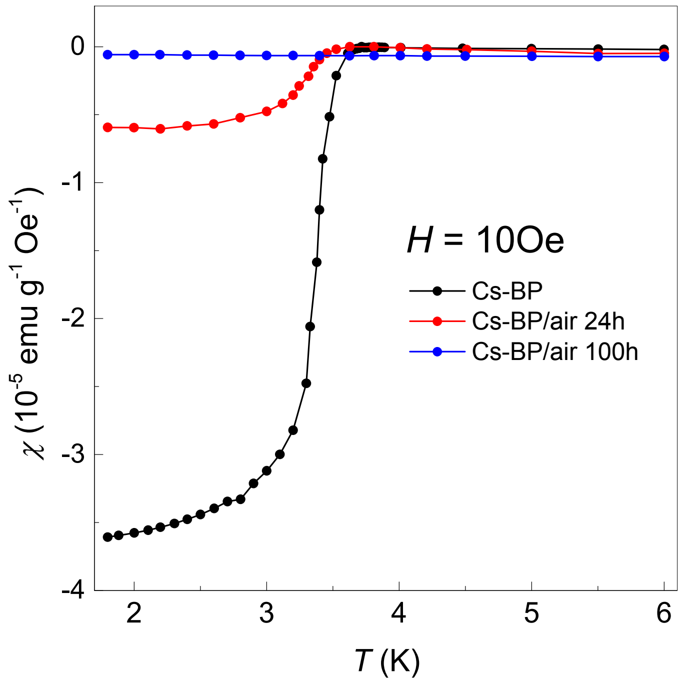

Supplementary Figure 3 | Air sensitivity and de-intercalation of superconducting black phosphorus. Shown is ZFC magnetic susceptibility, $\chi(T)$, of a Cs-intercalated BP sample before (black symbols) and after exposure to air for 24 hours (red symbols) and for 100 hours (blue symbols).

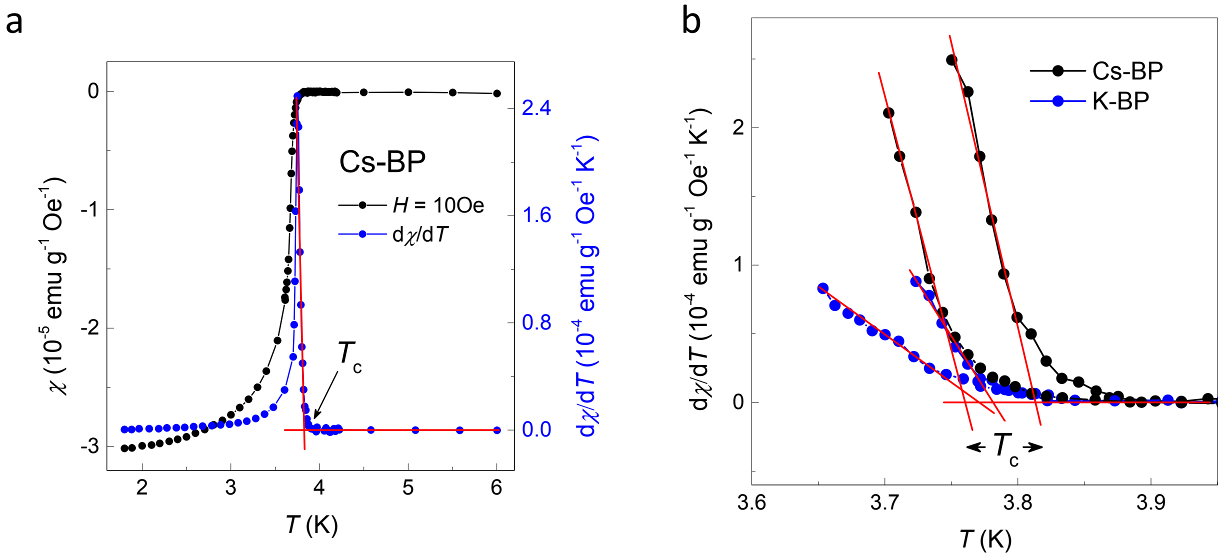

Supplementary Figure 4 | Determination of the critical temperature, T_c. (a) ZFC magnetic susceptibility, $\chi(T)$, for Cs-intercalated BP (black symbols). Blue symbols show the corresponding numerical derivative $d\chi/dT$. T_c is determined as the temperature corresponding to a sharp increase in $d\chi/dT$ and is given by a crossing point of the linear extrapolations of the corresponding parts of the curve. **(b)** Examples of T_c's for different samples intercalated with the same metal. The spread of T_c's for the same intercalant is similar to that for different intercalating metals (cf. inset in Fig. 2a in the main text) and limits our accuracy in finding the exact T_c.

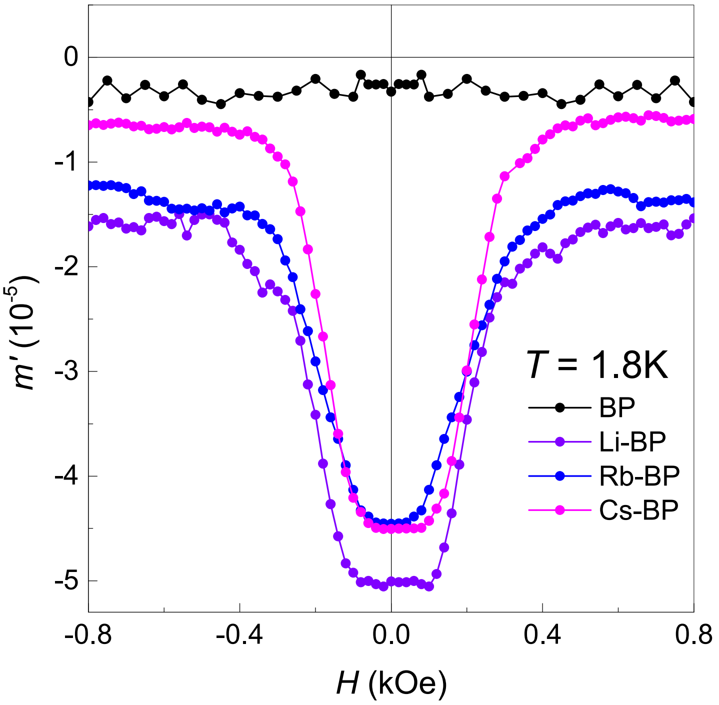

Supplementary Figure 5 | Magnetic-field dependent ac susceptibility of Li, Rb and Cs intercalated BP. Shown is the real part of ac susceptibility, m', versus H at T = 1.8K.

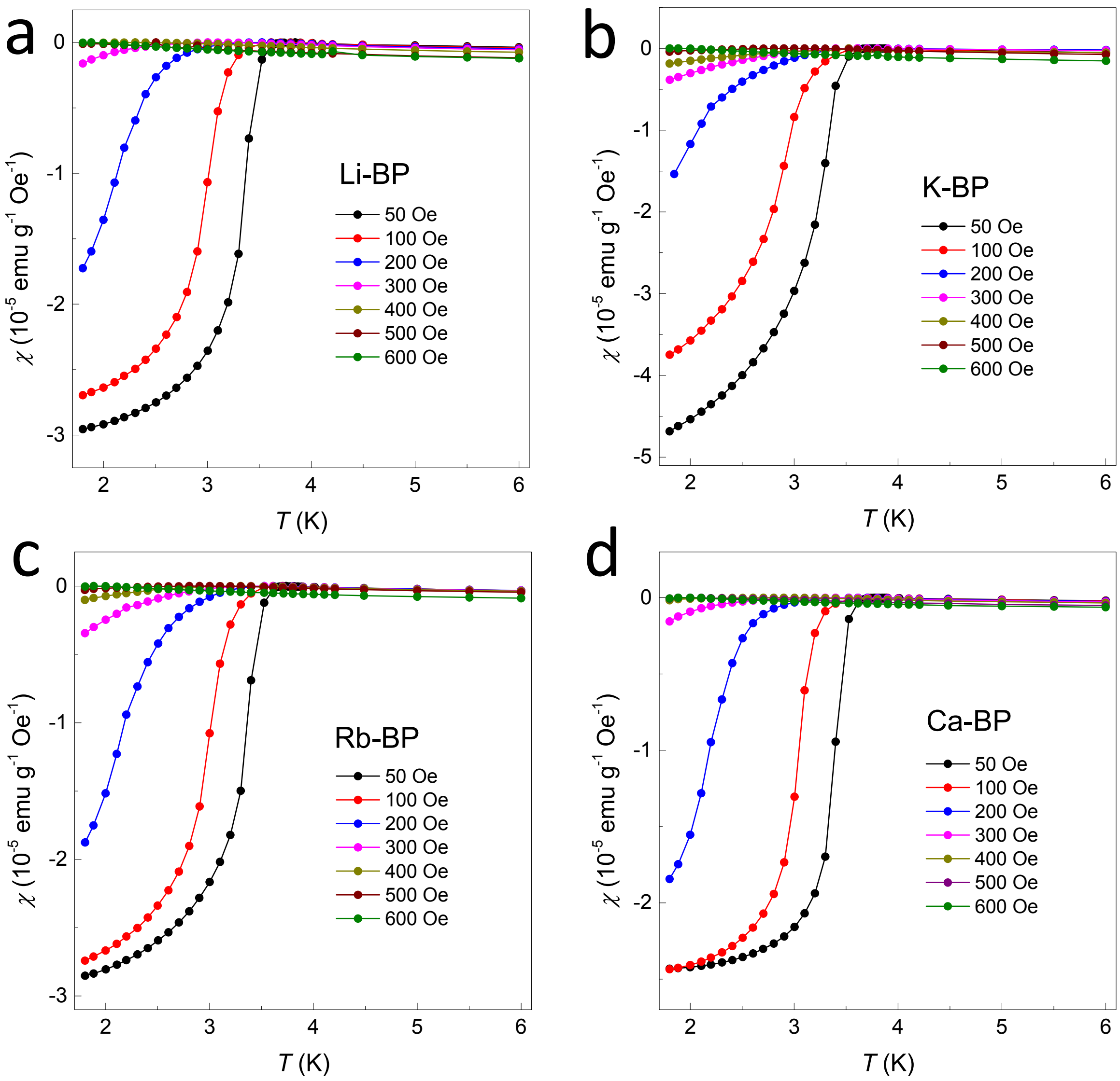

Supplementary Figure 6 | Suppression of superconductivity by magnetic field in intercalated BP. (a-d) ZFC dc susceptibility, $\chi = M/H$, as a function of temperature at different magnetic fields, H, for Li-, K-, Rb- and Ca-intercalated BP, respectively.

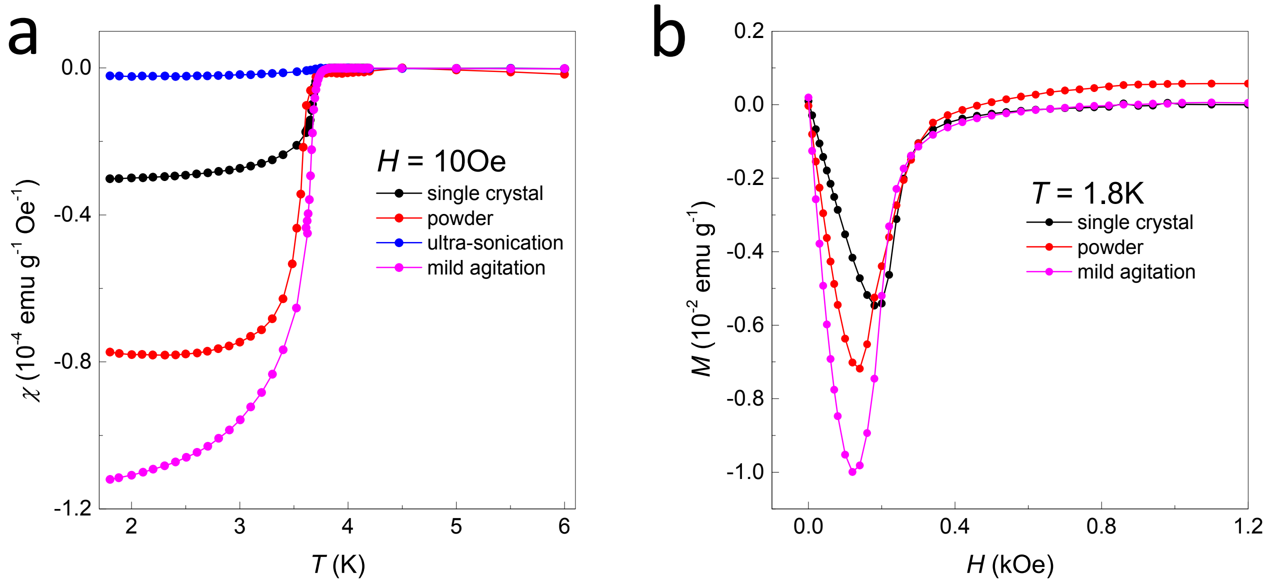

Supplementary Figure 7 | Increasing the superconducting fraction in intercalated BP. Field-dependent magnetization, $M(H)$, and temperature-dependent dc susceptibility, $\chi(T)$, for BP intercalated with Cs under different conditions (see Methods for details). Black symbols correspond to the magnetic response of a large, ~3×5×0.3 mm BP crystal intercalated with Cs. Red symbols correspond to intercalation of BP powder (a large crystal ground into powder prior to intercalation). Magenta and blue symbols correspond to the superconducting response of a large crystal that was broken up into many smaller and thinner crystals during intercalation by subjecting the liquid-ammonia solution to mild agitation and ultra-sonication, respectively. While mild agitation led to an increase of the superconducting fraction to ~10%, ultra-sonication resulted in suppression of superconductivity, presumably due to excessive damage to the BP crystal.

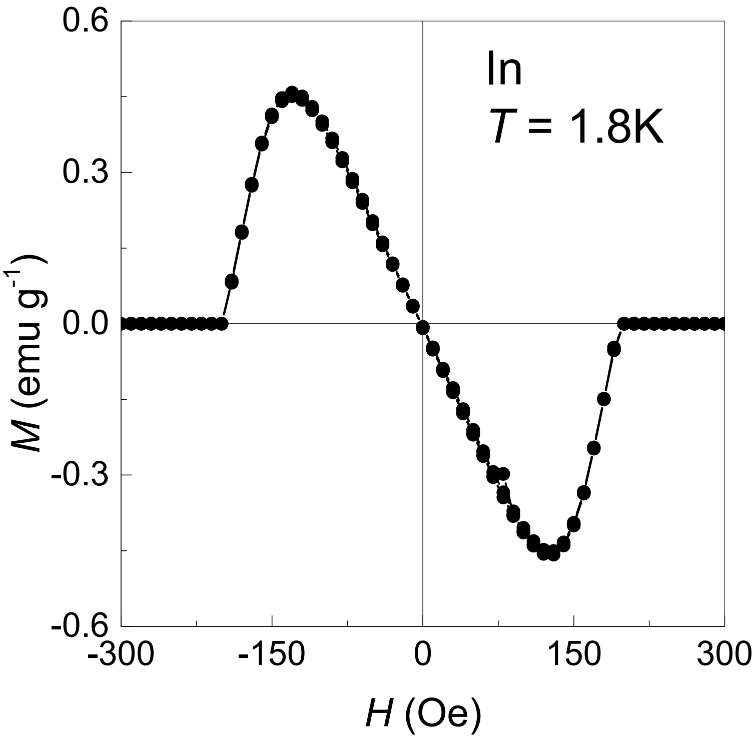

Supplementary Figure 8 | Magnetic-field dependent magnetization of a typical type-I superconductor. Shown is dc magnetization, *M,* as a function of magnetic field, *H*, at *T* = 1.8K for an indium (In) sample. The curves measured from -300 to 300 Oe and from 300 to -300 Oe overlap, due to the absence of flux pinning.

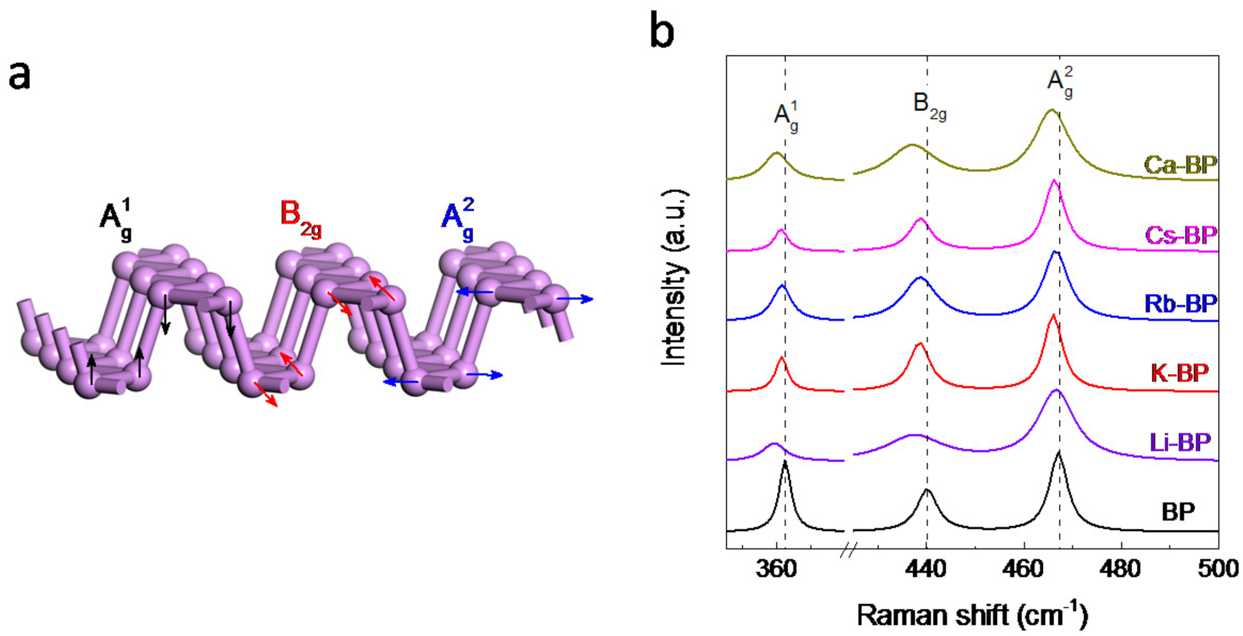

Supplementary Figure 9| Raman spectra for pristine and intercalated black phosphorus. (a) Schematic representation of the atomic displacements corresponding to Raman-active modes in BP. **(b)** Raman spectra for pristine and metal-intercalated black phosphorus (see labels). Dashed lines indicate the positions of the A_g^1, B_{2g} and A_g^2 Raman peaks for pristine BP.
